# Clinical manifestations of COVID-19 versus other upper respiratory tract infections in pediatric patients

**DOI:** 10.15537/smj.2023.44.1.20220439

**Published:** 2023-01

**Authors:** Manar M. Alzahrani, Abdulaziz K. Alaraifi, Lama H. Aldosari, Leen O. Hijazi, Fahad A. Alsaab

**Affiliations:** *From the College of Medicine (Alzahrani), King Saud bin Abdulaziz University for Health Sciences, from the Division of Otolaryngology-Head and Neck Surgery (Alaraifi, Hijazi, Alsaab), Department of Surgery, King Abdulaziz Medical City, Ministry of National Guard Health Affairs, Riyadh, and from the Department of Urology (Aldosari), King Fahad University Hospital, Alkhobar, Kingdom of Saudi Arabia.*

**Keywords:** COVID-19, URTI, taste, manifestations, children

## Abstract

**Objectives:**

To explore the differences between COVID-19 and upper respiratory tract infections (URTI) in the pediatric population, emphasizing smell and taste disturbances.

**Methods:**

A case-control study included 468 patients, 234 with COVID-19 (cases) and 234 with URTI (controls) at a tertiary hospital, Riyadh, Saudi Arabia, from 2020-2021. Patients with bacterial URTI, lower tract respiratory infections, and speech or developmental delays were excluded. Statistical analysis was carried out using Statistical Analysis System, 9.2 version. A *p*-value of ≤0.05 was considered significant.

**Results:**

The male-to-female ratio was almost equal, with a mean age of 9.90±2.34. Multivariable logistic regression analysis showed that a change in taste significantly increases the probability of COVID-19 by 21.98 times. On the other hand, sore throat (81.5%), dyspnea (63.5%), nasal obstruction (72.7%), and otalgia significantly (74.8%) decrease the likelihood of COVID-19.

**Conclusion:**

Taste disturbances increase the probability of COVID-19 infections, whereas sore throat, dyspnea, nasal obstruction, and otalgia increase the likelihood of other URTIs. The described differences might aid physicians in their differential diagnosis and treatment during the pandemic.

**C**oronavirus disease-19 (COVID-19), which is caused by severe acute respiratory syndrome coronavirus-2 (SARS-CoV-2), is an emerging infectious disease that has become a global pandemic causing a major public health concern since the outbreak of the pandemic in early 2020.^1,2^ According to World Health Organization (WHO), there have been over 500 million confirmed cases of COVID-19 globally, whereas the number of confirmed cases in Saudi Arabia exceeded 700,000 by the middle of 2022.^3,4^

The other rhinoviruses causing upper respiratory tract infections (URTIs) might mimic COVID-19 in its clinical presentation.^2^ More than 40% of preschool and 30% of school-age children present with flu-like symptoms during the influenza season.^5^ Therefore, it is challenging to differentiate them from pediatric patients with COVID-19, as they commonly present with only mild symptoms compared to adult patients.^5,6^

Published reports of adults with COVID-19 showed various symptoms, including fever, headache, cough, dyspnea, and smell and taste disturbances. According to Lee et al,^1^ 15.3% of adult patients reported anosmia and ageusia as early manifestations. Moreover, Beltrán-Corbellini et al^7^ reported anosmia to be significantly more frequent in COVID-19 patients (39.2%) than in those infected with influenza (12.5%) in the adult population. Therefore, smell and taste disturbances are crucial in differentiating COVID-19 from other rhinoviruses in adults. However, pediatric patients usually present asymptomatically or with mild symptoms compared to adults, with rarely reported smell and taste disturbances.^5,8^ As per Mak et al^6^ case series, only 3 pediatric patients were reported to have anosmia or ageusia.

Although there have been multiple studies addressing COVID-19 and other rhinoviruses in adults since the outbreak of the pandemic, limited data exist to compare the clinical presentation of COVID-19 to that of other URTIs in the pediatric population. Therefore, this study aims to explore the differences in the clinical presentation between both diseases in pediatric patients, emphasizing smell and taste disturbances; which might aid physicians in their differential diagnosis and assist in early detection and isolation during the pandemic.^5,9^

## Methods

This case-control study was carried out at the Pediatric Otorhinolaryngology Department at King Abdullah Specialized Children’s Hospital, Riyadh, Saudi Arabia. The medical records of 700 patients with COVID-19 and 500 patients with URTI from January 2020 to January 2021 were reviewed using a non-probability sampling technique. We enrolled all male and female pediatric patients (aged 6-14 years) diagnosed with either viral URTI or COVID-19. Patients with bacterial URTIs, lower respiratory tract infections, and developmental delays that would hinder them from reporting their symptoms were excluded. The asymptomatic patients with COVID-19, diagnosed based on active screening, were also excluded. The included patients’ diagnoses were made in accordance with interim recommendations from WHO based on their clinical presentation, then confirmed by carrying out a real-time reverse transcriptase-polymerase chain reaction test on nasopharyngeal and oropharyngeal swabs. Those who tested positive were enrolled in COVID group, whereas those who tested negative were enrolled in URTI group. Controls were matched with cases by age and gender using Propensity Score Matching (PSM) with a 1:1 ratio. Eventually, we included 2 comparable groups of 234 patients with URTI as controls and 234 patients with COVID-19 as cases.

The patient’s demographics, comorbidities, and clinical characteristics were extracted from National Guard Health Affairs electronic medical records, then the patients were contacted via phone calls at least 14 days after the initial visit and assessed subjectively using a comprehensive data sheet that included all commonly reported flu-like symptoms. The purpose of the phone calls was to document the undocumented symptoms and the newly developed ones within 14 days from the diagnosis day.

The institutional review board at King Abdullah International Medical Research Centre approved this study (study number: NRC21/003/01), and consents were obtained from parents of the included pediatric patients to be enrolled in this study.

### Statistical analysis

The data were entered and cleaned using Microsoft excel 2019 and analyzed using Statistical Analysis System (SAS^®^), version 9.2 for Mac. Continuous variables were summarized and reported as means and standard deviations (SDs), then compared across study groups using the T-test. Categorical variables were expressed as percentages and compared across the 2 study groups using the Chi-square test. A *p*-value of <0.05 was considered significant. The significant variables were further included in the multivariable regression model to identify the unique contribution of each variable.

## Results

The study included 468 patients, 234 with COVID-19 and 234 with URTI. The age and gender distribution of the 2 groups were relatively equal, with a mean age of 9.90±2.34 years. Regarding co-morbidities, the URTI group has a significantly higher incidence of asthma (23.1% vs. 12%, *p*=0.002) and allergic rhinitis (28.2% vs. 5.1%, *p*=0.000) compared to the COVID group. The demographics and clinical characteristics of all patients are summarized in Table 1.

**Table 1:** - Demographics and clinical characteristics of all study groups.

Characteristics	Overall (N=468)	COVID-19 (n=234)	URTI (n=234)	*P*-values
Age, mean±SD	9.9±2.34	10.05±2.34	9.75±2.34	0.167
***Age groups***				
6-10 years	259 (55.3)	119 (50.9)	140 (59.8)	0.051
11-14 years	209 (44.7)	115 (49.1)	94 (40.2)	
***Gender***				
Males	239 (51.1)	117 (50.0)	122 (52.1)	0.64
Females	229 (48.9)	117 (50.0)	112 (47.9)	
History of allergic rhinitis: itching and sneezing	78 (16.7)	12 (5.1)	66 (28.2)	0.000*
History of asthma	82 (17.5)	28 (12.0)	54 (23.1)	0.002*

Values are presented as numbers and precentages (%).

^*^ *P*-value of <0.05 was considered significant. COVID-19: coronavirus disease-19, URTI: upper respiratory tract infection, SD: standard deviation

Table 2 shows the clinical manifestations of the patients who presented with either disease. Fever was documented as the most frequent symptom in both groups; however, it appears at a higher rate in patients with URTI compared to the COVID group (82.1% vs. 65%, *p*=0.000). Similarly, sore throat (73.9% vs. 30.8%), cough (54.7% vs. 34.2%), rhinorrhea (45.3% vs. 27.8%), nasal obstruction (39.3% vs. 14.1%), dyspnea (33.3% vs. 12.4%), otalgia (17.5% vs. 4.7%), and otorrhea (5.1% vs. 0.4%) are significantly more common in URTI group in comparison to COVID group (*p*<0.05). On the other hand, smell (20.1%) and taste (24.4%) disturbances are more common in patients with COVID-19 (*p*<0.05). There was no statistically significant difference in the prevalence of headache between the 2 groups (*p*>0.05).

**Table 2:** - Clinical manifestations of coronavirus disease-19 and *upper respiratory tract infection* in pediatric patients.

Symptoms	Overall (N=468)	COVID-19 (n=234)	URTI (n=234)	*P*-values*
Fever	344 (73.5)	152 (65.0)	192 (82.1)	0.000
Sore throat	245 (52.4)	72 (30.8)	173 (73.9)	0.000
Cough	208 (44.4)	80 (34.2)	128 (54.7)	0.000
Rhinorrhea	171 (36.5)	65 (27.8)	106 (45.3)	0.000
Nasal obstruction	125 (26.7)	33 (14.1)	92 (39.3)	0.000
Headache	161 (34.4)	71 (30.3)	90 (38.5)	0.064
Dyspnea	107 (22.9)	29 (12.4)	78 (33.3)	0.000
Otalgia	52 (11.1)	11 (4.7)	41 (17.5)	0.000
Otorrhea	13 (2.8)	1 (0.4)	12 (5.1)	0.002
Change in taste	70 (15.0)	57 (24.4)	13 (5.6)	0.000
Change in smell	69 (14.7)	47 (20.1)	22 (9.4)	0.001

Values are presented as numbers and precentages (%).

^*^ *P*-value of <0.05 was considered significant. COVID-19: coronavirus disease-19, URTI: upper respiratory tract infection

Only 5 symptoms continued to be significant in the multivariable logistic regression analysis, as shown in Table 3. It showed that a change in teste significantly increases the probability of COVID-19 by 21.98 times (odds ratio=21.983, *p*=0.00). On the other hand, sore throat (81.5%), dyspnea (63.5%), nasal obstruction (72.7%), and otalgia (74.8%) significantly decrease the likelihood of COVID-19 (*p*≤0.05).

**Table 3:** - Statistically significant symptoms in logistic regression analysis.

Symptoms	*P*-values*	Odds ratios	95% confidence interval
Change in taste	0.000	21.983	7.455-64.826
Sore throat	0.000	0.185	0.113-0.302
Dyspnea	0.001	0.365	0.199-0.670
Nasal obstruction	0.000	0.273	0.140-0.533
Otalgia	0.005	0.252	0.096-0.664

^*^ *P*-value of <0.05 was considered significant. The reference category is: coronavirus group.

Table 4 compares smell and taste changes between COVID and URTI groups. Our study sample was divided into 2 age subgroups: 6-10 years old and 11-14 years old. Taste disturbances were significantly more prevalent in the COVID group compared to the URTI group in both age subgroups (*p*<0.05). The prevalence of smell disturbances was significantly higher in the COVID group than in the URTI group, in the older subgroup only (*p*=0.008). There was no statistically significant difference in the prevalence of smell disturbances between COVID and URTI groups in the younger subgroup (*p*>0.05).

**Table 4:** - Change in smell and taste in coronavirus disease-19 versus *upper respiratory tract infection* according to age.

Symptoms	6-10 years (n=259)			11-14 years (n=209)		
	COVID-19	URTI	*P*-values	COVID-19	URTI	*P*-values
Change in taste	23 (19.3)	9 (6.4)	0.002*	34 (29.6)	4 (4.3)	0.000*
Change in smell	20 (16.8)	13 (9.3)	0.152	27 (23.5)	9 (9.6)	0.007*

Values are presented as numbers and precentages (%).

^*^ *P*-value of <0.05 was considered significant. COVID-19: coronavirus disease-19, URTI: upper respiratory tract infection

## Discussion

Severe acute respiratory syndrome coronavirus-2 has infected approximately 500 million people worldwide since the pandemic, including more than 700 thousand cases in Saudi Arabia. Limited data exist on the global or local prevalence of pediatric cases specifically. With mean mortality of 7%, nearly 6 million people have died of COVID-19 worldwide, with over 9 thousand deaths reported in Saudi Arabia alone.^3,4^ These reported deaths have exceeded the estimated annual deaths from seasonal influenza.^10,11^

In the current study, fever was documented as the most common reported symptom in both groups; however, its presence does not increase or decrease the likelihood of COVID-19 infection. Sore throat, dyspnea, nasal obstruction, and otalgia were found to increase the probability of URTI. Otologic symptoms, such as otalgia and otorrhea, are rarely reported in the COVID group compared to the URTI group. The presented differences showed that the URTI group has a broader range of symptoms than the COVID group. Similarly, Pormohammad et al^12^ carried out a systemic review comparing clinical findings of COVID-19 to influenza type A and B. They concluded that runny nose, dyspnea, sore throat, and rhinorrhea were less frequent in COVID-19 than in influenza type A and type B.^12^ On the other hand, Chandrasekaran et al^2^ compared the presentation of COVID-19 and influenza in adults in France. The most common symptoms of both disorders were reported to be fever, fatigue, cough, myalgia, and arthralgia (>50% of cases), with no statistically significant differences in the frequencies of each complaint between the 2 groups.^2^

The sudden onset of anosmia and dysgeusia can guide physicians and the medical community to suspect COVID-19 early in the disease course.^13,14^ In the present study, taste disturbances significantly increased the probability of COVID-19 by 21.98 times. On the other hand, smell disturbances do not have an impact on the likelihood of COVID-19 infection. Kaye et al^15^ found that anosmia was the main presenting symptom in 26.6% of COVID-19. Anosmia usually appears 5 days after the onset of the other COVID-19 symptoms. It may persist for up to 28 days with a mean duration of 7 days, whereas the average time of anosmia with URTI lasts less than 3 days.^16^ In the pediatric population, smell and taste dysfunction lasted between 3-13 days in the 3 cases reported by Mak et al.^6^ A total loss of smell was noted in all 3 patients and 2 of them showed changes in taste.^6^ The pathogenesis of olfactory dysfunction in COVID-19 is attributed to sensory receptor damage, an olfactory cranial nerve lesion, or a central neural lesion. The cellular receptor angiotensin-receptor enzyme 2, which is primarily expressed in human airway epithelia and lung parenchyma, mediates the entry of SARS-COV-2 into human host cells, which explains the development of these symptoms in COVID-19 patients.^2,17^

The current study showed that smell disturbances are more common in the older group (11-14 years old) in comparison to the younger group (6-10 years old). Similar to Hijazi et al,^18^ olfactory dysfunction was noted more in older children (11-15 years old) with a prevalence of 8.8% than in the younger ones (7-10 years old) with a prevalence of 3.3%. This could be attributed to the milder disease course and receptor immaturity in children. Furthermore, it was demonstrated in the literature that younger children are less reliable in detecting gustatory and olfactory disturbances.^19,20^

In adults, COVID-19 has a broad spectrum of presentations, with otorhinolaryngological symptoms being the most common ones.^21^ A systemic review by Neto et al^22^ addressed 13 studies discussing the clinical manifestations of COVID-19 infection. It showed that low to moderate fever was the most frequent symptom, detected in more than half of the participants. Respiratory symptoms were identified in all studies, with cough, productive or non-productive, emerging as the second most prevalent symptom among adult patients infected with COVID-19.^22^ Another systemic review by da Rosa Mesquita et al^23^ included 152 publications with a total of 41,409 individuals from 23 countries. The 3 main symptoms were fever (57.9%), cough (54.2%), and dyspnea (30.6%), while the least frequent symptom was hemoptysis (1.7%).^23^

Coronavirus disease-19 manifests milder in children, which is attributed to a less pronounced inflammatory response. The occurrence of this disease pattern appears to correlate with age inversely.^24^ A systemic review by Uzunoglu et al^25^ included 25 articles covering 2446 pediatric patients showed that the overall rate of the asymptomatic patients was 24.8%, mild disease was observed in 40.7%, mild pneumonia in 27%, severe pneumonia in 5.3%, and only 3% of the patients had critical severity. The most commonly reported manifestations were fever (61.7%), cough (53.2%), diarrhea or nausea (16.8%), and lymphopenia (15%). Laboratory and radiological findings of COVID-19 in pediatrics are not specific, except for lymphopenia and ground glass opacity on radiographs, which may have a diagnostic value.^25^

### Study limitations

The present study was carried out in a tertiary healthcare center, which affects the study’s generalizability. It was a retrospective study; thus a susceptibility to collection bias on exposures could happen. Another limitation is the lack of objective assessment of the patients due to the isolation measures implemented during the pandemic; therefore, phone call interviews were carried out to collect the data and follow up with the patients. Hence, prospective multicenter studies with objective evaluations are recommended in the future studies.

In conclusion, taste disturbances increase the probability of COVID-19 infections, whereas sore throat, dyspnea, nasal obstruction, and otalgia increase the likelihood of other URTIs. Smell disturbances do not increase or decrease the probability of COVID-19 in pediatric patients. The described differences might aid physicians in their differential diagnosis and treatment during the pandemic.
